# Stroke in mitral valve prolapse: risk factors and left atrial function in cryptogenic versus non-cryptogenic ischemic subtypes

**DOI:** 10.3389/fneur.2023.1058697

**Published:** 2023-07-25

**Authors:** Francesca Calicchio, Lisa J. Lim, Danielle Cross, Dwight Bibby, Qizhi Fang, Karl Meisel, Nelson B. Schiller, Francesca N. Delling

**Affiliations:** ^1^Department of Medicine (Cardiovascular Division), The Lundquist Institute at Harbor—University of California-Los Angeles Medical Center, Torrance, CA, United States; ^2^Department of Medicine (Cardiovascular Division), University of California-San Francisco, San Francisco, CA, United States; ^3^Department of Neurology, Lancaster General Health Neuroscience Institute, Lancaster, PA, United States; ^4^Department of Neurology, McLaren Northern Michigan Neurosciences, Petoskey, MI, United States

**Keywords:** stroke, mitral valve prolapse (MVP), left atrial function, left atrial function index, echocardiography, cryptogenic stroke

## Abstract

**Background and purpose:**

Mitral valve prolapse (MVP) has been associated with an increased risk of ischemic stroke. Older age, thicker mitral leaflets, and significant mitral regurgitation (MR) leading to atrial fibrillation have been traditionally considered risk factors for ischemic stroke in MVP. However, specific risk factors for MVP-stroke subtypes are not well defined. The aim of this study is to evaluate clinical and echocardiographic parameters, including left atrial (LA) function, in MVP with cryptogenic (C) vs. non-cryptogenic (NC) stroke.

**Methods:**

In this case-control matched study, MVPs were identified in consecutive echocardiograms obtained after a stroke from January 2013 to December2016 at the University of California, San Francisco. MVP was defined as leaflet displacement ≥2 mm in the parasternal long-axis view at end-systole. Age/gender matched MVPs without stroke and healthy controls without MVP were also identified. We analyzed LA end-systolic/diastolic volume index, emptying fraction (LAEF), function index (LAFI), and global longitudinal strain in all MVPs and controls. We also measured left ventricular (LV) volume indexes, mass index, ejection fraction (EF), degree of MR and leaflet thickness.

**Results:**

We identified a total of 30 MVPs (age 70 ± 12, 50% females) with stroke (11 with C- and 19 with NC-stroke), 20 age/gender matched MVPs without a stroke and 16 controls. MVPs without stroke had lower BMI, less hypertension but more MR (≥moderate in 45% vs. 17%), more abnormal LA function (lower LAEF, LAFI) and larger LV volumes/mass (all *p* < 0.05) when compared to MVPs with stroke. Leaflet thickness was overall mild (<3 mm) and similar in the 2 groups. Within the MVP stroke group, NC-stroke had higher BMI, more hypertension and more atrial fibrillation compared to C-stroke. In the variables tested, patients with C-stroke did not differ from controls.

**Conclusions:**

MVP-related MR may be protective against stroke despite abnormal LA function. Risk of NC-stroke in MVP is related to common stroke risk factors rather than mitral valve leaflet thickness. The etiology of C-stroke in MVP warrants further studies.

## Introduction

Mitral valve prolapse (MVP) is a common valvular disorder occurring in 2 to 3% of the general population (1–3). It is characterized by fibro-myxomatous degeneration of one or both leaflets of the MV with systolic displacement into the left atrial chamber (4). MVP is the most important cause of primary mitral regurgitation (MR) requiring surgical correction (5, 6, 11, 12). Over the years, MVP has been the object of attention due to its prognostic implications, including its association with cerebral ischemic events. However, the relationship between MVP and stroke remains controversial. Discrepancies exist across studies (7–10) with some investigations-mainly conducted in tertiary care centers - showing a higher rate of ischemic stroke or transient ischemic attacks in individuals affected by MVP (8, 9, 31). The mechanism mediating this relationship appeared to be mainly the increased incidence of AF in patients affected by MVP even though older age, MV thickness and severity of MR also appeared to be contributing factors. A few authors have also described familial patterns of MVP complicated by neurological events, strengthening the link between the two conditions (13). Other studies enrolling younger patients have not confirmed the association between MVP and ischemic stroke (7, 8). The relationship between MVP and cerebrovascular ischemic events prompts even more attention if we consider that about 25% of all ischemic strokes remains cryptogenic in nature (14), without an identifiable source of cardio-embolism, atherosclerosis of the large arteries or disease of the small arteries. Moreover, most of the existing literature on MVP and stroke has focused only on non-cryptogenic (NC) stroke with most of the studies not addressing determinants of cryptogenic (C) cerebrovascular ischemic events or differences with NC stroke (25). In addition, prior research studies have not investigated left atrial (LA) function as a possible mediator of stroke among MVP subjects with and without severe MR or atrial fibrillation (AF). Because specific risk factors for MVP- stroke subtypes remain poorly defined, we sought to investigate both clinical and echocardiographic features, including LA emptying fraction (LAEF), left atrial function index (LAFI) and strain associated with C- and NC- stroke in a cohort of patients with MVP.

## Methods

### Study population

In our case-control study, cases were defined by a concomitant diagnosis of MVP and stroke. Specifically, the presence of MVP was assessed in consecutive echocardiograms obtained after a stroke from January 2013 to December 2016 at the University of California, San Francisco. MVPs were evaluated by two independent cardiologists blinded to the stroke subtypes. Age/gender matched MVPs without stroke and age/gender matched healthy controls were also included in the study. Controls had normal physical examination, were free from cardiovascular disease and had no comorbidities. Exclusion criteria for the study group were as follows: presence of a patent foramen ovale (PFO), history of endocarditis, prior mitral valve surgery, or suboptimal image quality. Baseline demographics and clinical data were collected for all study participants. Each study subject underwent comprehensive neurological and echocardiographic examination as detailed below. The study protocol was approved by the Institutional Review Board of the University of California, San Francisco. All participants included in the study gave written informed consent.

### Baseline characteristics

Baseline demographics and clinical data were obtained from electronic medical records and included the following: age, sex, race/ethnicity, smoking status, systolic and diastolic blood pressure, body mass index (BMI), co-existing hypertension and diabetes mellitus, medical history of atrial fibrillation. Presence of atrial fibrillation was documented based on 12 lead electrocardiogram (ECG) recording, inpatient telemetry, 48-hours Holter, or 2-week event monitor (Ziopatch).

### Neurological evaluation

Study subjects underwent full neurological evaluation including: (i) identification of risk factors for cerebrovascular disease through history taking, (ii) detailed physical exam, (iii) blood tests comprising lipid panel and coagulation profile, and (iv)imaging studies including transcranial Doppler imaging, brain computed tomography and/or magnetic resonance and/or contrast angiography and vascular Doppler imaging. Acute ischemic stroke was defined as evidence of altered circulation involving the cerebral hemispheres, the brain stem or the cerebellum occurring less than one week prior to admission and causing neurological symptoms persisting >24 h (14). Transient ischemic attack (TIA) was defined as a temporary brain deficit caused by vascular insufficiency and determining symptoms that resolve completely within 24 hrs. Stroke was classified as C and NC upon definition of etiology and in accordance with the American Stroke Association guidelines (14). C-stroke was defined as a brain infarction of undetermined etiology, not clearly attributable to a definite cardio-embolic source, large artery atherosclerosis, or small artery disease nor to hematological disorders, nonatherosclerotic vasculopathies or hypercoagulable stateaccording to the TOAST (trial of ORG 10172 in acute stroke treatment) classification (14). NC-stroke was defined as stroke caused by one of the conditions listed above. The final adjudication of type of stroke was based on chart review of clinical notes and testing by one neurologist (DC) and confirmed by a second neurologist (KM).

### Echocardiographic evaluation

**Figure 1:**
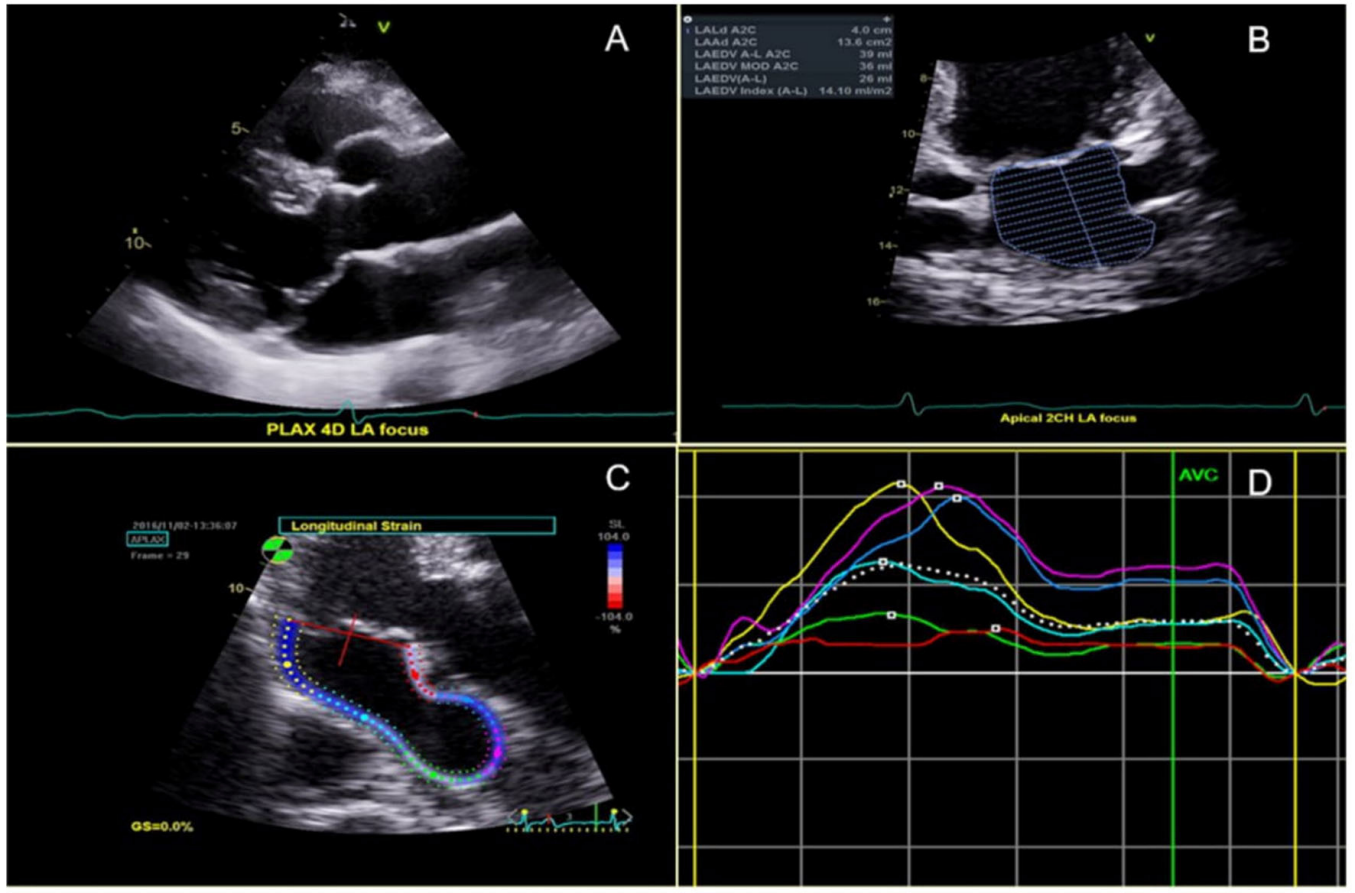
**(A)** Bileaflet MVP shown in the parasternal long axis view. **(B)** Left atrial (LA) end-diastolic volume in the 2-chamber view. **(C)** 2D-Segmental LA strain from a zoomed apical long axis view; and **(D)** corresponding segmental LA strain graph.

Comprehensive transthoracic echocardiography was performed according to current guidelines from the American Society of Echocardiography (ASE) (15) using a variety of commercially available cardiovascular ultrasound machines as part of standard clinical evaluation. Two-dimensional cine loops of parasternal long- and short-axis, and apical two-, three-, and four-chamber views were obtained in all MVPs and controls. All images and measurements were acquired from standardized windows and stored. The digitally stored echocardiographic images were analyzed with offline software and reviewed by 2 senior cardiologists (FND and NBS). MVP was defined as systolic leaflet displacement ≥2 mm beyond the mitral annulus in the parasternal or apical long-axis views (Figure 1A). Leaflet involvement was categorized into anterior, posterior or bileaflet. MV leaflet thickness was measured at end-diastole in the same views as the leading to the trailing edge of the thickest area of the mid-portion of the leaflet, excluding focal areas of thickness and chordae. Anterior and posterior leaflet thickness measurements were averaged for the analysis. For each MVP patient, degree of MR was classified as mild, moderate or severe according to the proposed ASE criteria (15). When quantitative assessment of MR was not available, its severity was based on visual estimation of the regurgitant jet. For patients in AF at the time of echocardiography, all measurements were calculated by averaging the data from five consecutive beats. LA end-systolic volume (LA ESV) and end-diastolic volume (LA EDV) were measured from the apical four- and two-chamber views using the Simpson's method, and were indexed to body surface area (BSA) to obtain LA ESVI and LA EDVI, respectively. The maximum LA volume was measured immediately prior to MV opening by tracing the inner border of the atrium, and excluding the area under the valve annulus, the atrial appendage, and the pulmonary veins (Figure 1B). The minimum left atrial volume was obtained at the time of MV closure. LA emptying fraction (LAEF) was calculated by applying the following formula: (LAEF = LA maximum volume–LA minimum volume/LA maximum volume × 100% (15). LAFI was calculated using the following formula: (LAFI = LAEF x LVOT VTI/LA max index) (16). The left ventricular outflow tract velocity time integral (LVOT VTI) was recorded by placing a 2 mm pulsed Doppler sample volume in the outflow tract below the aortic valve. Global peak systolic longitudinal left atrial strain (LA GLS) was obtained in all MVPs and controls. It was measured at the end of the reservoir phase by using the average of the strain values obtained from atrial segments in 2-chamber (*n* = 6 segments) and 4-chamber (*n* = 6 segments) views (28). The average of the two was used for the analysis (Figures 1C, D). LV end-diastolic and end-systolic volumes were measured and indexed for BSA (LV EDVI/LV ESVI). The LV mass was calculated using the formula (LV mass = 0.8 {1.04 [([LVEDD + IVSd + PWd]3 - LVEDD3)]} + 0.6) and indexed for BSA to obtain LV mass index (LVMI). LV ejection fraction (LVEF) was calculated using the Simpson's method.

### Statistical analysis

Results are presented as mean ± standard deviation (SD) for continuous variables and as counts and percentages (%) for discrete variables. Normally distributed continuous variables were compared using the one-way analysis of variance (ANOVA) test or Student's *t*-test for three and two-group comparisons, respectively. The association between categorical variables was determined using Fisher's exact test given the small sample size for some comparisons. Due to the low number of MVP cases with a history of ischemic stroke overall, and within the C- and NC-stroke subcategories, we did not run a multivariable model testing the association of MVP with stroke (well established in prior literature) (8–10, 13). Two-tailed p values <0.05 were considered statistically significant. All statistical analyses were performed using standard statistical software (SAS V. 9.4, SAS Institute, Cary, North Carolina, USA).

## Results

### Baseline demographic and clinical characteristics

We identified a total of 30 MVPs with stroke, 20 age/gender matched MVPs without a stroke and 16 healthy controls. Baseline clinical data of the 3 groups is summarized in Table 1. MVPs with stroke (*n* = 30) were predominantly male (*n* = 13, 43%) and Caucasians. Their mean age was 70 ± 12 years. MVP with stroke had a higher BMI and a higher prevalence of hypertension, although the proportion of AF was similar compared to MVPs without stroke. Among the 5 MVPs with stroke and AF (17%), AF was paroxysmal in 2. None of the controls had AF. AF prevalence was ascertained through 12-lead EKG in 6 (50%), and Ziopatch, 48-h Holter or telemetry in the remaining 6 (50%). The majority were non-smokers (Table 1). Among the MVPs with stroke, 11 were cryptogenic (C-stroke) and 19 were non-cryptogenic (NC-stroke). Etiology of NC-stroke was as follows: cardio-embolic in 5 (26%), small vessel disease in 3 (16%), other determined etiology in 9 (47%). Two (10%) of the NC-strokes were TIAs. NC-stroke had more hypertension and AF compared to C-stroke (Table 1). In all the clinical variables tested, patients with C-stroke did not differ from controls (all *p* > 0.05).

**Table 1:** Baseline clinical and echocardiographic characteristics between MVPs with stroke, MVPs without stroke and healthy controls.

	**MVPs with stroke (*N* = 30)**	**MVPs w/o stroke (*N* = 20)**	**Controls (*N* = 16)**	***P***
Age	70 ± 11	71 ± 13	65 ± 5	0.8
Male	13 (43%)	9 (45%)	8 (50%)	0.9
BMI	26 ± 5	22 ± 4	25 ± 5	0.009
Hypertension	24 (80%)	9 (45%)	4 (25%)	0.01
Diabetes	5 (17%)	2 (10%)	5 (31%)	0.4
Smoke	Not smoker, 22 (73%); Smoker or former smoker, 6 (20%)	Not smoker, 11 (55%); Smoker or former smoker, 9 (45%)	N/A	0.1
AF	5 (17%)	7 (35%)^*^	0	N/A
≥moderate MR	5 (17%)	9 (45%)^*^	0	N/A
Prolapsing leaflets	Anterior, 6 (20%) Posterior, 12 (40%) Bi-leaflet, 12 (40%)	Anterior, 3 (15%)^*^ Posterior, 7 (35%) Bi-leaflet, 10 (50%)	0	N/A
Mitral leaflet thickness (mm)	2.6 ± 0.7	2.9 ± 0.7	2.5 ± 0.7	0.2
LVEF (%)	63 ± 9	61 ± 11	65 ± 5	0.4
LV EDVI (ml/m2)	47 ± 15	61 ± 16	52 ± 16	0.005
LV ESVI (ml/m2)	17 ± 7	23 ± 9	18 ± 8	0.003
LVMI (g/m2)	70 ± 15	85 ± 12	60 ± 14	0.001
LA EF (%)	57 ± 7	41 ± 10	57 ± 6	0.001
LA EDVI (ml/m2)	26 ± 9	46 ± 18	12 ± 5	< 0.001
LA ESVI (ml/m2)	12 ± 5	28 ± 14	26 ± 9	< 0.001
LAFI	0.55 ± 0.29	0.22 ± 0.16	0.54 ± 6.7	< 0.001
LA global strain (%)	29 ± 7	28 ± 9	28 ± 10	0.3

Continuous variables were compared using the one-way analysis of variance (ANOVA) test. Categorical variables were compared using Fisher's exact test. ^*^p = 0.04 when comparing the presence of significant mitral regurgitation (MR) among subjects with mitral valve prolapse (MVP) with vs. without stroke; P was NS when comparing type of prolapsing leaflets and prevalence of atrial fibrillation (AF) between the same 2 groups.

### Echocardiographic measurements

MVPs with stroke differed significantly from MVPs without stroke with regards to several echocardiographic characteristics (see Table 1). MVPs without stroke had more MR (≥moderate in 45% vs. 17%, more abnormal LA function (lower LAFI and LAEF), and larger LV volumes and mass (all *p* < 0.05) compared to MVPs with stroke and to controls. Leaflet thickness (overall mild and <3 mm) and the pattern of prolapsing leaflets (anterior, posterior or both) did not differ significantly between MVPs with and without stroke (Table 1). LA GLS was also similar in the 2 groups. Within the MVP stroke group, NC-stroke had more abnormal LA function (lower LAFI) compared to C-stroke. Leaflet thickness and degree of MR were similar (Table 2). LA GLS was numerically higher in MVP with C-stroke compared to MVP with NC-stroke, although the comparison did not reach statistical significance (Table 2). In the echocardiographic variables tested, patients with C-stroke did not differ from controls (all *p* > 0.05).

**Table 2:** Baseline clinical and echocardiographic characteristics between MVP with cryptogenic (C) vs. non-cryptogenic (NC) stroke.

	**MVP with C-stroke (*N* = 11)**	**MVP with NC-stroke (*N* = 19)**	***p***
Age	70 ± 8	71 ± 14	0.9
Male	4 (36%)	9 (47%)	0.7
BMI	24 ± 4	27 ± 6	0.1
Hypertension	6 (55%)	16 (84%)	0.01
Diabetes	2 (18%)	3 (16%)	0.9
AF	0 (0%)	5 (26%)	N/A
MR degree	≥moderate MR, 2 (18%)	≥moderate MR, 3 (16%)	0.9
Prolapsing leaflets	Anterior, 3 (27%) Posterior, 5 (45%) Bi-leaflet,3 (27%)	Anterior, 4 (21%) Posterior, 10 (53%) Bi-leaflet, 5 (26%)	0.9
Mitral leaflet thickness	2.3 ± 0.6	2.3 ± 0.5	0.7
LVEF (%)	67 ± 6	61 ± 9	0.1
LV EDVI (ml/m2)	43 ± 14	49 ± 15	0.2
LV ESVI (ml/m2)	15 ± 6	18 ± 6	0.07
LVMI (g/ml2)	67 ± 13	72 ± 16	0.4
LA EF (%)	57 ± 9	49 ± 12	0.07
LA EDVI (ml/m2)	24 ± 6	31 ± 13	0.1
LA ESVI (ml/m2)	11 ± 4	17 ± 12	0.09
LAFI	0.56 ± 0.20	0.37 ± 0.17	0.009
LA global strain (%)	35 ± 11	28 ± 10	0.1

## Discussion

In the present study, we demonstrate that, despite abnormal LA function (lower LAEF and LAFI), significant MVP-related MR may be protective against stroke. Traditional risk factors such as obesity and hypertension rather than increased leaflet thickness are associated with ischemic stroke, particularly NC-stroke, in MVP. Indeed, high blood pressure and hypertension are known to play a crucial role in the development of systemic atherosclerosis and its clinical manifestations in cerebrovascular disease (26). In our study we utilized detailed neurological assessment and, for the first time, we incorporated standard and advanced echocardiographic parameters of LA function to better understand determinants of ischemic stroke and its subtypes. The focus of our study were patients with MVP and stroke who were then compared with MVP without stroke and healthy controls.

### Traditional risk factors for stroke in MVP

Our findings of greater severity of MR in MVP without stroke corroborate previous mechanistic and epidemiological studies highlighting a protective effect of severe MR (17–23). Specifically, Karatasakis et al. demonstrated that the presence of significant MR in patients with rheumatic mitral valve disease reduced the incidence of spontaneous echocontrast, thrombi and embolic events in relation to a “washing” effect of the MR jet (17). Movsowitz et al. retrospectively analyzed 427 patients undergoing transesophageal echocardiography (TEE) for different reasons including pre-op assessment, cardiac anomalies, bacterial endocarditis, aortic dissection, source of embolism and assessment of LV function. In this study, the authors found that significant MR was protective against both left atrial echo contrast and thrombus formation (17). More recently, Cresti et al. demonstrated that the incidence of left atrial thrombus was significantly lower in adult patients with severe functional MR assessed by TEE in patients with paroxysmal or persistent AF/flutter undergoing electrical cardioversion (22). Finally, Nakagami et al. studied 290 patients with non-rheumatic AF and found that MR was protective against stroke, especially in those patients who had LA enlargement (20). A more recent study by Yang et al. showed that patients with less than moderate MR due to MVP exhibit early LV and LA remodeling, however this does not predict MR progression or mortality (21). In our study, we add to prior knowledge by demonstrating that in MVP patients MR is protective even in the presence of abnormal LA function, which was not previously evaluated in the setting of MVP and stroke. Discrepant data exists regarding the protective effect of MR. Avierinos et al. reported an association between MVP, degree of MR and cerebrovascular ischemic events in 777 subjects enrolled in the Olmsted County community. The main mechanism relating MVP and stroke was thought to be the development of AF as a consequence of severe MR. They concluded that older age, degree of MR, leaflet thickness and LA diameter were all predictors of long-term AF and, in turn, of stroke (9). Another study by Bisson et al. showed a neutral effect of MR on stroke risk (28). We postulate that the discrepancy between these investigations and the many others demonstrating a lower risk of stroke with severe MR (including ours) may be related the high prevalence of patients with AF but no MR (up to 90% in one study) (29), where the protective effect of MR may have been mitigated by other determinants of AF such as diastolic dysfunction or hypertension. Moreover, our study included MVP patients with C-stroke, where AF is less prevalent, hence the protective effect of MR was likely not obscured by the development of AF.

Although degree of MR was greater in the no stroke group, there was no difference between the two MVP groups with regards to leaflet thickness or leaflet involvement (bileaflet vs. monoleaflet). Our results suggest that the causal relationship between MVP and cerebral ischemic events may not reside in intrinsic features of the diseased mitral valve (such as leaflet thickness) nor in the extent/pattern of involvement of the leaflets, but rather in atherosclerotic disease driven by hypertension and higher BMI. The importance of traditional atherosclerotic factors was particularly evident in the NC-stroke patients. Avierinos et al. (9) found that older age was a predictor of ischemic neurological events in MVP patients in the community. In our study, we did not observe a significant age difference between MVPs with stroke and without stroke nor between the C-stroke and NC-stroke (see Tables 1, 2). However, the average age in all groups was 70 years, indicating that the overall sample in our study was not young. Hence, the effect of aging could not be assessed due to lack of a comparative group of younger individuals.

Finally, regarding the role of AF in our study group, we did not detect any difference in the prevalence of AF between MVPs with and without stroke (*p* = 0.2). However, when stratifying by stroke subtype, we confirmed that all subjects with AF belonged to the NC-stroke subgroup, highlighting the known importance of AF as a determinant of ischemic stroke in general and in the MVP population.

### LA function and stroke in MVP

Although LA GLS is expected to be lower with higher degrees of MR (31), we did not find a significant difference in LA GLS between MVP patients without stroke (and more “protective” MR) vs with stroke. We hypothesize that other causes of abnormal LA GLS such as hypertension and obesity, more prevalent in the stroke group, may have contributed to a decrease in LA GLS, hence “balancing out” values between the stroke and no stroke groups. Lower LA GLS has been shown to correlate with LA fibrosis in patients with and without mitral valve disease in histological studies (30–33). Although one small study describes LA strain patterns in MVP vs. rheumatic heart disease and suggests that peak atrial longitudinal strain (PALS) may be lower in rheumatic mitral valve disease compared to MVP (24), our study is the first to investigate the role of LA GLS (rather than PALS) in MVP and stroke. Interestingly, when we analyzed the subgroups C-stroke vs NC-stroke, LA GLS was numerically lower (albeit not meeting statistical significance) in NC-stroke, in conjunction with other parameters of LA dysfunction such as decreased LAFI in this subgroup. Even outside of the MVP context, LAFI was the best predictor of cardio-embolic stroke (25) in a study by Ferikh et al. In this study, despite the high prevalence of AF (74%), there was no significant correlation between LAEF and stroke. Altered LA parameters, in particular LAFI, and an abnormal “LA substrate” may be at the root of cardio-embolic stroke in the majority of patients (regardless of MVP status). LAFI, a composite measure of atrial structural and functional remodeling, is inversely associated with age, hypertension and cardiovascular disease (27). Conversely, lower rates of hypertension and obesity in MVPs with C-stroke in our study, and, as a consequence, higher LAFI in this subgroup compared to NC-stroke suggest that LA dysfunction may not be the primary determinant of C-stroke in MVP. Normal LA function in C-stroke was corroborated by similar clinical characteristics and LA function parameters to controls. Overall, larger sample studies are needed to confirm these findings and better understand the etiology of C-stroke.

### Study limitations

First, our study was carried out at one center on a relatively small sample of patients. Therefore, some data comparisons may not be significant. The small sample size was mainly related to the low number of patients with MVPs and concomitant stroke who were identified in our Echocardiography Laboratory. Second, the assessment of AF in our study was based on 12-lead EKG, 48-h Holter and 2-week event monitoring. Because continuous heart rhythm monitoring was not feasible in our study population and because some of the patients did not have an event monitor (Ziopatch), self-limiting episodes of AF may have been missed. In addition to this, because of the relatively small number of patients with AF, we did not reach sufficient statistical power for the analysis of this variable. Therefore, we could not accurately comment on the role of AF type/duration on the occurrence of cerebral vascular events. Further studies with larger number of patients and longer follow-up duration are warranted.

## Conclusion

MVP-related MR may be protective against stroke despite abnormal LA function. Risk of NC-stroke in MVP is related to common stroke risk factors, rather than to mitral valve leaflet thickening. The etiology of C-stroke in MVP warrants further research.

## Data availability statement

The raw data supporting the conclusions of this article will be made available by the authors, without undue reservation.

## Ethics statement

The studies involving human participants were reviewed and approved by UCSF Ethic Committee. The patients/participants provided their written informed consent to participate in this study.

## Author contributions

The authors have contributed to the manuscript as follows: FD, FC, and KM made a substantial contribution to the concept or design of the article. DB, NS, and QF contributed to acquisition of data and data analysis. FC, LL, DC, and FD contributed to the interpretation of the data, drafted the manuscript, and critically revised it. All authors reviewed and approved the manuscript for publication.
